# Immunogenicity and Tolerability after Two Doses of Non-Adjuvanted, Whole-Virion Pandemic Influenza A (H1N1) Vaccine in HIV-Infected Individuals

**DOI:** 10.1371/journal.pone.0036773

**Published:** 2012-05-21

**Authors:** Heimo Lagler, Katharina Grabmeier-Pfistershammer, Veronique Touzeau-Römer, Selma Tobudic, Michael Ramharter, Judith Wenisch, Guido Andrés Gualdoni, Monika Redlberger-Fritz, Theresia Popow-Kraupp, Armin Rieger, Heinz Burgmann

**Affiliations:** 1 Department of Medicine I, Division of Infectious Diseases and Tropical Medicine, Medical University of Vienna, Vienna, Austria; 2 Department of Dermatology, Division of Immunology, Allergy and Infectious Diseases, Medical University of Vienna, Vienna, Austria; 3 Department of Tropical Medicine, University of Tübingen, Tübingen, Germany; 4 Department of Virology, Medical University of Vienna, Vienna, Austria; Saint Louis University, United States of America

## Abstract

**Background:**

During the influenza pandemic of 2009/10, the whole-virion, Vero-cell-derived, inactivated, pandemic influenza A (H1N1) vaccine Celvapan® (Baxter) was used in Austria. Celvapan® is adjuvant-free and was the only such vaccine at that time in Europe. The objective of this observational, non-interventional, prospective single-center study was to evaluate the immunogenicity and tolerability of two intramuscular doses of this novel vaccine in HIV-positive individuals.

**Methods and Findings:**

A standard hemagglutination inhibition (HAI) assay was used for evaluation of the seroconversion rate and seroprotection against the pandemic H1N1 strain. In addition, H1N1-specific IgG antibodies were measured using a recently developed ELISA and compared with the HAI results. Tolerability of vaccination was evaluated up to one month after the second dose. A total of 79 HIV-infected adults with an indication for H1N1 vaccination were evaluated. At baseline, 55 of the 79 participants had an HAI titer ≥1∶40 and two patients showed a positive IgG ELISA. The seroconversion rate was 31% after the first vaccination, increasing to 41% after the second; the corresponding seroprotection rates were 92% and 83% respectively. ELISA IgG levels were positive in 25% after the first vaccination and in 37% after the second. Among the participants with baseline HAI titers <1∶40, 63% seroconverted. Young age was clearly associated with lower HAI titers at baseline and with higher seroconversion rates, whereas none of the seven patients >60 years of age had a baseline HAI titer <1∶40 or seroconverted after vaccination. The vaccine was well tolerated.

**Conclusion:**

The non-adjuvanted pandemic influenza A (H1N1) vaccine was well tolerated and induced a measurable immune response in a sample of HIV-infected individuals.

## Introduction

A new swine-origin, triple-reassortant influenza A (H1N1) virus that emerged in Mexico in late March 2009 began to spread rapidly through human-to-human transmission outside the usual influenza season [1]–[3]. On June 11th, 2009, the World Health Organization raised the influenza pandemic alert to the highest level (level 6) as human influenza A (H1N1) cases were reported worldwide in 74 countries [4]. The pandemic virus was antigenically and genetically unrelated to human seasonal influenza virus, and former seasonal influenza vaccines appeared not protective. Reports of severe respiratory failure associated with this strain, particularly in young persons, forced the rapid implementation of a vaccine and resulted in development of several pandemic anti-influenza A H1N1 2009 vaccines to be distributed around the world [1]–[3]. In October 2009, in addition to various adjuvanted pandemic H1N1 vaccines, the European Medicines Agency (EMA) licensed an inactivated whole-virion, Vero-cell-derived pandemic H1N1 influenza A/California/07/2009 vaccine without adjuvant. This vaccine was based on an earlier H5N1 mock-up vaccine [3]. In Austria, the Federal Ministry of Health selected this vaccine for use during the national pandemic vaccination campaign from November 2009 to March 2010 and therefore it was the only available pandemic vaccine in Austria during the whole of the pandemic period. At this time international guidelines from the Centers for Disease Control and Prevention recommended vaccination [5] particularly for immunocompromised individuals, since underlying medical conditions such as immunosuppression appeared to predispose for infection with H1N1 [2]. Immunosuppressed patients are at increased risk of both greater morbidity due to influenza infection [6] and lower immune response rates to vaccination [7]. During the pandemic it was therefore recommended that at least all high-risk HIV-positive individuals and their close contacts should receive pandemic influenza vaccine as one of the most effective preventive measures or at least to mitigate the severity of illness and impact of the disease [8].

According to the Committee for Medicinal Products for Human Use (CHMP) at the EMA [9], the following serological assessments should be considered in adult subjects aged between 18 and 60 years, and at least one of the assessments should meet the indicated requirements: (1) 70% of subjects should show seroprotection after vaccination (hemagglutination inhibition (HAI) antibody titers ≥1∶40); (2) 40% of subjects should show seroconversion (≥4-fold increase in HAI antibody titer after vaccination and post-vaccination titers ≥1∶40); (3) the increase in geometric mean titers (GMTs) after vaccination should be >2.5-fold. For adults >60 years the CHMP requirements are: 60% of subjects achieving seroprotection, >30% of subjects showing seroconversion or significant (≥4-fold) increase in HAI antibody titers, and a GMT increase after vaccination >2-fold. An HAI antibody titer of 1∶40 is associated with a 50% reduction in risk of illness in a susceptible adult population [10], [11].

It is well established that whole-virion vaccines are more immunogenic than conventional split-virion vaccines [12]–[14], and this particular whole-virion vaccine has shown good immunogenicity in mice [15]. Reports at the beginning of the vaccination program indicated promising immunogenicity and tolerability in the healthy population [3]. The objective of our study was to determine immunogenicity and tolerability after first and second doses of this unique adjuvant-free pandemic influenza A (H1N1) vaccine in a population of HIV-infected individuals during the pandemic because responses to this vaccine were uncertain at that time.

## Methods

### Participants

HIV-infected persons of both sexes, willing to be vaccinated, were recruited between November 2009 and March 2010 at Vienna General Hospital, Austria, during their routine visits to the HIV outpatient department. We excluded individuals with known cases of influenza A (H1N1) infection and previous recipients of any other new pandemic 2009 influenza A (H1N1) vaccine. Blood samples for determination of the virus-specific antibody response were obtained at baseline before vaccination (t_0_) and after each vaccination (t_1_ and t_2_). Inclusion criteria included HIV serology confirmed by western blot and PCR, absence of fever (body temperature ≤37.5°C), and a fully completed and signed standardized information sheet that provided information on the new pandemic vaccine Celvapan® (Baxter, Vienna, Austria) and recorded the medical history of the individual. Clinical, laboratory, and HIV-related data were extracted from patients' charts. Detailed vaccination histories were also collected. Adverse effects and influenza-specific symptoms were recorded during indicated visits or by phone one month after each vaccination.

### Vaccine details

The inactivated whole-virion vaccine Celvapan® (Baxter, Vienna, Austria) [16] was derived from cultured Vero cells and supplied in multidose vials, each 0.5 ml dose containing 7.5 µg of hemagglutinin from influenza A/California/07/2009 (H1N1). Virions had been inactivated with formaldehyde and UV irradiation and purified on a sucrose density gradient. The finished product was a suspension for intramuscular injection without added adjuvant or preservatives such as antibiotics. Celvapan® was the only adjuvant-free pandemic H1N1 vaccine licensed in Europe [3], [17] and the only available pandemic 2009 influenza A H1N1 vaccine in Austria during the pandemic of 2009/2010 ( = study period). The vaccination schedule consisted of an injection into the deltoid muscle on days 0 and 21 or later.

### Laboratory methods

The immune response to vaccination was assessed using a standard HAI assay and an H1N1-specific immunoglobulin G (IgG) ELISA before and after each vaccination, as previously described [18]. Blood samples collected as part of routine care were used for the tests, and all sera were continuously stored at −80°C before testing in duplicate by a reference laboratory (Bonostix GmbH & Co. KG, Kornwestheim, Germany).

The standardized HAI [19] assay employed chicken red blood cells and influenza A/H1N1pdm virus A/California/7/2009 obtained from the National Institute for Biological Standards and Control (UK). The following immunogenicity endpoints were calculated from individual HAI antibody titers: the proportions of seroprotection (HAI antibody titers ≥1∶40 after vaccination), seroconversion (HAI antibody titer prevaccination <1∶10 and postvaccination ≥1∶40 *or* prevaccination ≥1.10 and a four fold increase or more postvaccination), and the ratio of GM of HAI antibody titers before and after vaccination [9].

In addition, specific IgG antibodies to pandemic H1N1 were determined using a commercial ELISA (Pandemic New Influenza A ELISA IgG/IgA Testkit, Genzyme Virotech GmbH, Rüsselsheim, Germany). According to the manufacturer's instructions, the presence of H1N1-specific antibodies is indicated by IgG antibodies >11units (U). Levels between 9 U and 11 U are counted as negative (below seroprotection limit). The described IgG test sensitivity and specificity were 95.7% and 95.0% respectively.

### Statistical analysis

Data of all participants (intention-to-treat population) were recorded on predesigned paper forms and checked manually before entry into a purpose-built computer database for analysis using a commercial software package (JMP 5.0, SAS, NC). Descriptive statistics were computed for patient demographics, seroconversion rates, and mean antibody titers. Differences in seroconversion rates were assessed using the χ^2^ test or Student's t-test as appropriate. The sample size of this study was estimated to detect a 60% difference in response rates between groups (β = 0.08) assuming 40% seroconversion and allowing for a 10% loss to follow up (n = 79). The significance level was set at α = 0.05 and no formal correction for multiple testing was performed.

### Ethical approval and compliance with national guidelines

We obtained formal ethical approval from the local ethics committee at the Medical University of Vienna (Austria). The committee waived the need for informed consent from individual participants of the study because blood collected for routine investigations was used for testing immune responses to vaccination and therefore no additional blood sampling was necessary. The study was classified as service evaluation.

Guidance from the Austrian Federal Ministry of Health was that all HIV-infected individuals should be considered at high risk for a severe course of 2009 pandemic influenza A (H1N1) and should therefore receive the vaccine Celvapan® (Baxter, Vienna, Austria) as one of the initial target groups and as a part of outpatient care. All data were collected and handled in accordance with the standards of Good Clinical Practice and Austrian regulatory requirements. The study was conducted in accordance with the principles of the Declaration of Helsinki.

## Results

Seventy-nine HIV-infected persons (19 women; median age 40 years, 95% CI 37–42 years; 7 individuals >60 years) were vaccinated, evaluated, and provided blood samples for serological analysis. Overall, 72 study participants (91%) completed the schedule of two vaccinations, seven people were vaccinated only once. Table 1 shows the demographic, clinical and laboratory characteristics of the study population. None had received high-dose immunosuppressive regimens, four women were pregnant at the time of vaccination. Median time intervals were as follows: between the first and second vaccinations 28 days (95% CI 26–31), between first vaccination and second blood sample 28 days (95% CI 27–32), between second vaccination and third blood sample 56 days (95% CI 51–70). Before vaccination, all 79 blood samples were available for serological testing. After the first vaccination 71 blood samples of 79 patients were available, and after the second vaccination 70 blood samples of 72 patients were available.

**Table 1:** All included HIV-positive individuals (n = 79) and the response to first intramuscular dose of the novel inactivated, non-adjuvanted, whole-virion pandemic influenza A (H1N1) vaccine detected by HAI and H1N1-specific IgG serology in 71 available blood samples.

	All included patients	Available blood samples after first vaccination n = 71			
		HAI		IgG ELISA	
		Seroconverters	Nonseroconverters	Seropositivity	Seronegativity
**N (n, %)**	79	22 (31%)	49 (69%)	17 (24%)	54 (76%)
**Sex, female (n, %)**	19 (24%)	8 (50%)	8 (50%)	4 (27%)	11 (73%)
**Age (years)^a^**	40 (37–42)	34 (29–38)	42 (39–45)	37 (32–42)	40 (37–43)
**HAI titer ≥1∶40 before vaccination (n, %)^b^**	55 (70%; 59–79%)	10 (21%; 12–35%)	37 (79%; 65–88%)	13 (27%; 17–41%)	35 (73%; 59–83%)
**age >60 years (n, %)^b^**	7 (9%; 4–17%)	0 (0%)	5 (100%; 57–100%)	0 (0%)	5 (100%; 57–100%)
**age ≤60 years (n, %)^b^**	72 (91%; 83–96%)	22 (33%; 23–45%)	44 (67%; 55–77%)	17 (26%; 17–37%)	49 (74%; 63–83%)
**AIDS criteria (CDC C3) (n, %)^b^**	11 (14%; 8–23%)	3 (30%; 11–60%)	7 (70%; 40–89%)	2 (22%; 6–55%)	7 (78%; 45–94%)
**Duration of HIV infection (months)^a^**	75 (61–89)	63 (38–89)	74 (57–91)	75 (46–104)	68 (52–84)
**HAART (n, %)^b^**	69 (87%; 78–93%)	19 (31%; 21–44%)	42 (69%; 56–79%)	16 (26%; 17–38%)	45 (74%; 62–83%)
**HIV RNA PCR load (log_10_copies/ml)^a^**	4.2 (3.6–4.4)	3.7 (<1.7–4.1)	4.3 (3.3–4.6)	3.7 (<1.7–4.1)	4.3 (3.4–4.5)
below limit of quantification (n, %)^b^	48 (61%; 50–71%)	14 (33%; 20–47%)	29 (67%; 53–80%)	10 (23%; 13–38%)	33 (77%; 62–87%)
50–1000 (n, %)^b^	12 (15%; 8–23%)	3 (30%; 19–73%)	7 (70%; 27–81%)	5 (50%; 19–73%)	5 (50%; 19–73%)
>1000 (n, %)^b^	19 (24%; 15–33)	4 (22%; 10–47%)	14 (78%; 52–90%)	2 (11%; 3–34%)	16 (89%; 66–97%)
**cd4 cell count (cells/µl)^a^**	502 (449–556)	483 (394–572)	489 (430–549)	439 (340–539)	508 (453–564)
<200 (n, %)^b^	3 (4%; 1–11%)	1 (50%; 9–91%)	1 (50%; 9–91%)	1 (50%; 9–91%)	1 (50%; 9–91%)
200–350 (n, %)^b^	18 (23%; 15–33%)	5 (29%; 13–53%)	12 (71%; 47–87%)	4 (25%; 10–49%)	12 (75%; 51–90%)
>350 (n, %)^b^	58 (73%; 63–82%)	17 (33%; 22–46%)	35 (67%; 54–78%)	13 (25%; 15–38%)	40 (75%; 62–85%)
Nadir cd4 cell count (nadir cell/µl)^a^	243 (203–282)	278 (201–355)	241 (190–294)	207 (120–295)	268 (219–317)
Duration of nadir cd4 cell count (months)^a^	40 (30–51)	25 (4–45)	47 (33–60)	46 (22–69)	39 (25–52)

**Figure 1:**
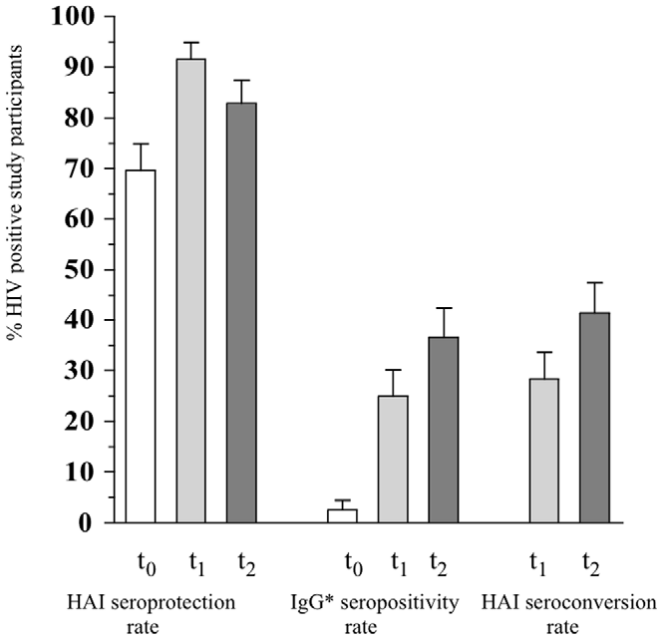
Comparison of three vaccination outcome parameters. 1) three columns left: HAI seroprotection rate (HAI antibody titers ≥1∶40); 2) three columns center: IgG* seropositivity rate (IgG*>11 U); 3) two columns right: HAI seroconversion rate (4-fold increase in HAI antibody titers to ≥1∶40); at three time points: t_0_, t_1_ and t_2_. Abbreviations: HAI, hemagglutination inhibition test; IgG*, pandemic H1N1-specific immunoglobulin G serology; t_0_, time before vaccination; t_1_, time after first vaccination; t_2_, time after second vaccination.

**Figure 2:**
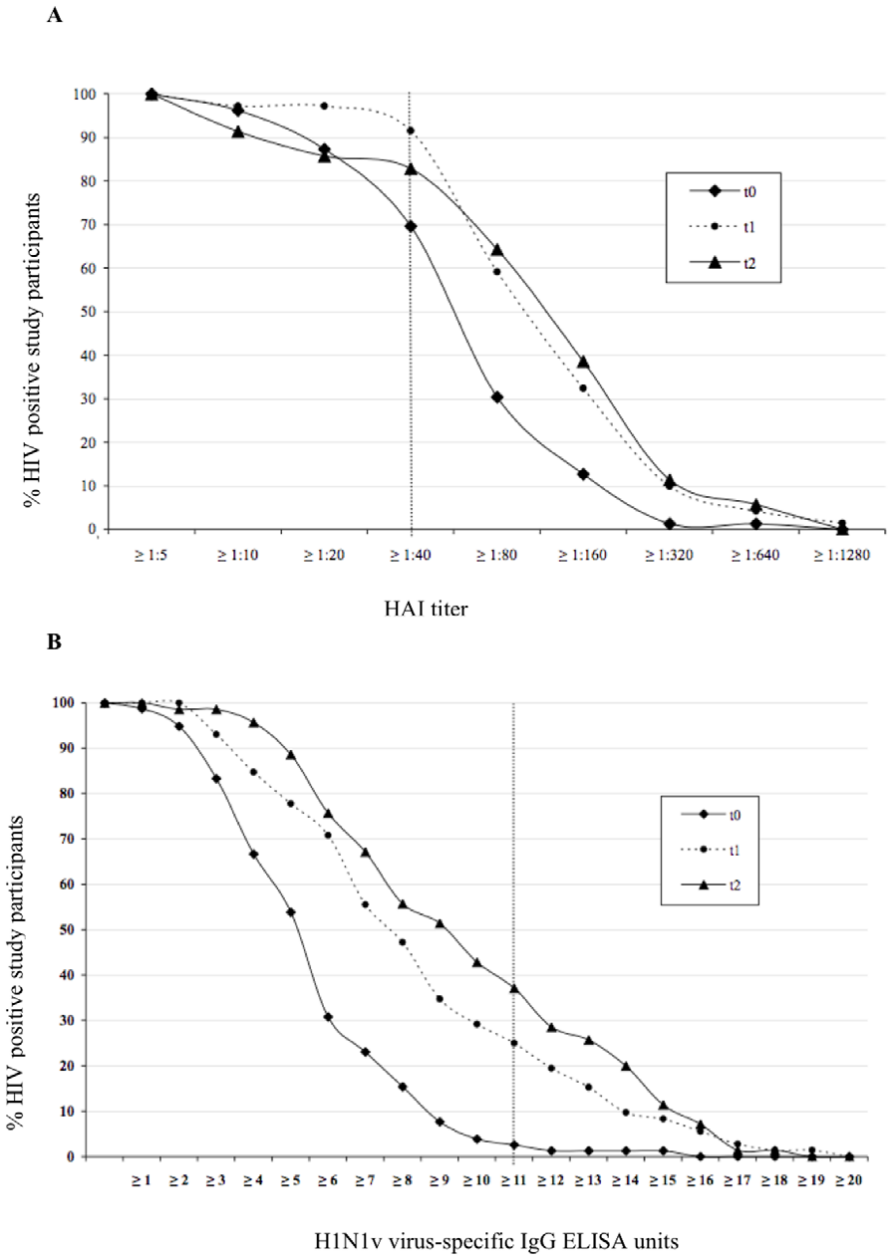
Reverse cumulative distribution curves. On HAI assay (Panel A) and H1N1 virus-specific IgG serology by ELISA (Panel B) before vaccination (t_0_), after the first vaccination (t_1_) and after the second vaccination (t_2_). Abbreviations: HAI, hemagglutination inhibition test; IgG, immunoglobulin G.

At baseline 55 (70%) of the participants (n = 79) already had HAI titers ≥1∶40 (>1∶40, n = 24, 30%). Among those ≤60 years, 48 (67%) of 72 had HAI titers ≥1∶40 (>1∶40, n = 19, 26%). In contrast, only two (3%) of 78 baseline serum samples were positive for H1N1 virus-specific IgG in the ELISA (Table 2, Figures 1 and 2). Following the first vaccination 65 (92%) of 71 serum samples and following the second vaccination 58 (83%) of 70 serum samples had an HAI titer ≥1∶40 (Table 2, Figures 1 and 2). Seroconversion was found in 22 (31%) of 71 patients after the first vaccination and increased to 29 (41%) of 70 patients after the second vaccination.

**Table 2:** Serological response, defined by seroprotection^a^, seroconversion^b^, GMT^c^ and GMT ratio^c^ and seropositivity^d^ in HIV-positive individuals before and after first and second intramuscular vaccination with the whole-virion, Vero cell-derived, inactivated, non-adjuvanted pandemic H1N1 influenza vaccine.

Timepoints	t_0_	t_1_	t_2_
Available blood samples	n = 79	n = 71	n = 70
**Seroprotection^a^**	55 (70%; 95%CI 59–79%)	65 (92%; 95%CI 83–96%)	58 (83%; 95%CI 72–90%)
**Geometric mean^c^**	40 (95%CI 7–237)	76 (95%CI 11–508)	70 (95%CI 6–793)
**Geometric mean ratio^c^ to baseline**	NA	1.9 (95%CI 0.2–20)	1.7 (95%CI 0.1–23)
**Seroconversion^b^**	NA	22 (95%CI 31%; 21–42%)	29 (95%CI 41%; 31–53%)
**Seropositive^d^**	2 (3%; 95%CI 1–9%)	18 (25%; 95%CI 16–35%)	26 (37%; 95%CI 27–49%)

Before vaccination the overall median HAI titer was 1∶40 (IQR 1∶20–1∶80), increasing to 1∶80 (IQR 1∶40–1∶160) after the first vaccination but not increasing further after the second dose. The median IgG ELISA values were 5.3 U (IQR 3.4–6.8) at baseline, increasing to 8 U (IQR 5.6–11) after the first vaccination and 9.6 U (IQR 6.3–13.1) after the second; both these were below the seroprotection limit and therefore counted as negative.

For individuals who seroconverted in the HAI assay the median titer after the second vaccination was 1∶160 (IQR 1∶80–1∶160) compared with 1∶40 (IQR 1∶20–1∶80) for nonconverters. ELISA testing, however, showed seropositivity in 18 of 72 (25%) patients after the first vaccination and in 26 of 70 (37%) after the second (Figure 1).

**Figure 3:**
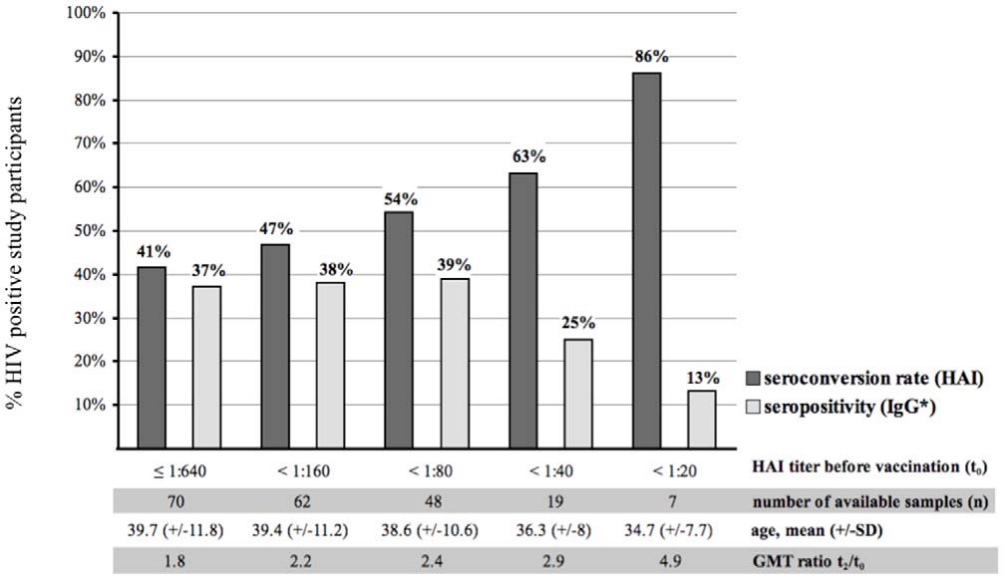
Correlation of HAI antibody titer before vaccination (t_0_), mean age of HIV-positive study participants, seroconversion rate (HAI), GMT ratio (HAI) and seropositivity (IgG* ELISA) after the second vaccination. Abbreviations: HAI, hemagglutination inhibition test; IgG*, pandemic H1N1-specific IgG, immunoglobulin G serology; t_0_, time before vaccination; t_2_, time after second vaccination; SD, standard deviation; GMT, geometric mean HAI titer.

The GMT measured by HAI assay was 76 after the first vaccination and 70 after the second dose (Table 2). The corresponding GMT ratios to baseline were 1.9 and 1.7 respectively. In the seroconverter group the GMT was 103 after the first vaccination and 152 after the second (corresponding GMT ratios 4.3 and 6.4). For nonconverters the GMT was 71 after the first vaccination and 56 after the second (corresponding GMT ratios to baseline 1.3 and 0.8). Among the 19 of 72 participants with baseline HAI antibody titers <1∶40, at least 79% (n = 15) developed protective antibodies and 63% (n = 12) seroconverted after the second vaccination. Among the individuals with an HAI baseline titer <1∶20 (n = 7), 86% (n = 6) developed protective HAI antibody titers and the same proportion seroconverted after the second dose (Figure 3).

In the HAI assay, seroconverters were younger than nonconverters, particularly those who converted after the first vaccination (34 vs. 42 years; p = 0.006) and had an HAI baseline titer <1∶40 (21% vs. 79%; p = 0.015). Furthermore, after the second vaccine dose the group ≤60 years seroconverted in the HAI assay more frequently (45% vs. 55%; p = 0.038) than the older age group >60 years. Detailed immunogenicity and statistical results in the different subgroups after the first dose of vaccine are summarized in Table 1 and after the second dose in Table 3.

**Table 3:** Response of HIV-positive individuals to second intramuscular dose of the inactivated, non-adjuvanted, whole-virion pandemic influenza A (H1N1) vaccine detected by HAI and H1N1-specific IgG serology in 70 available blood samples.

	Available blood samples after second vaccination n = 70			
	HAI		IgG ELISA	
	Seroconverters	Nonseroconverters	Seropositivity	Seronegativity
**N (n, %)**	29 (41%)	41 (59%)	26 (37%)	44 (63%)
**Sex, female (n, %)**	6 (37.5%)	10 (62.5%)	6 (40%)	9 (60%)
**Age (years)** a	37 (33–42)	42 (38–46)	40 (35–45)	40 (36–44)
**HAI titer ≥1∶40 before vaccination (n, %)** b	17 (33%; 22–47%)	34 (67%; 53–78%)	21 (42%; 29–56%)	29 (58%; 44–71%)
**age >60 years (n, %)** b	0 (0%)	6 (100%; 61–100)	2 (33%; 10–70)	4 (67%; 30–90%)
**age ≤60 years (n, %)** b	29 (45%; 34–57%)	35 (55%; 43–66%)	24 (38%; 27–50%)	40 (63%; 50–73%)
**AIDS criteria (CDC C3) (n, %)** b	1 (17%; 3–56%)	5 (83%; 44–97%)	2 (29%; 8–64%)	5 (71%; 36–92%)
**Duration of HIV infection (months)** a	67 (44–90)	80 (60–99)	88 (63–112)	66 (47–84)
**HAART (n, %)** b	27 (46%; 34–58%)	32 (54%; 42–66%)	25 (40%; 29–53%)	37 (60%; 47–71%)
**HIV RNA PCR load (log_10_copies/ml)** a	3.6 (<1.7–4.0)	4.4 (3.5–4.7)	3.2 (<1.7–3.6)	4.4 (3.6–4.7)
below limit of quantification (n, %)^b^	16 (38%; 25–53%)	26 (62%; 47–75%)	19 (45%; 31–60%)	23 (55%; 40–69%)
50–1000 (n, %)^b^	6 (60%; 31–83%)	4 (40%; 17–69%)	5 (45%; 17–69%)	6 (55%; 31–83%)
>1000 (n, %)^b^	6 (33%; 18–61%)	12 (67%; 39–82%)	2 (12%; 3–36%)	15 (88%; 63–97%)
**cd4 cell count (cells/µl)** a	494 (403–584)	533 (457–609)	510 (415–605)	531 (458–604)
<200 (n, %)^b^	2 (67%; 21–94%)	1 (33%; 6–79%)	2 (67%; 21–94%)	1 (33%; 6–79%)
200–350 (n, %)^b^	5 (38%; 18–64%)	8 (62%; 35–82%)	4 (33%; 14–61%)	8 (67%; 39–86%)
>350 (n, %)^b^	23 (43%; 30–56%)	31 (57%; 44–70%)	20 (36%; 25–50%)	35 (64%; 50–75%)
Nadir cd4 cell count (nadir cell/µl)^a^	265 (198–331)	236 (180–292)	209 (141–278)	273 (220–326)
Duration of nadir cd4 cell count (months)^a^	29 (12–46)	49 (35–63)	41 (23–59)	42 (28–56)

Abbreviations: HAI, hemagglutination inhibition test; IgG, immunoglobulin G;

a median (95% confidence interval),

b (%; 95% confidence interval).

Pregnancy (n = 4) and comorbidity such as hepatitis C (n = 7) or diabetes (n = 3) at the time of vaccination did not correlate with the three measured immunogenicity parameters. A subgroup of 47 patients were systematically asked about seasonal influenza vaccination received during the previous three years or other vaccinations in the past 12 months. The immunization rate was in general low (2009/10 n = 9; 2008/09 n = 3; 2007/08 n = 4; other vaccinations, n = 2) and there was no correlation with HAI titer at baseline or the three immunogenicity parameters (data not shown).

Local tolerability was excellent. A painful induration at the injection site for one day was reported for only one of 151 vaccinations (79 first doses, 72 second doses). Systemic side effects were rare. One patient developed generalized pruritus after the first dose and was not vaccinated again. After the second dose, two patients developed symptoms of fatigue considered possibly related to the vaccination by the investigators. The only serious adverse event necessitating hospital admission was a fatal fulminant hepatitis induced by isoniazid as part of TB therapy; this event was considered unrelated to the vaccination. Four patients had some type of upper respiratory illness with coughing as the main symptom. Two of them developed coughing and subfebrile body temperature <38°C after the first vaccination (one case 1 day after vaccine, one case 2 weeks after vaccine). The other two patients developed coughing and fever >38°C after the second vaccination (one case 1 day after vaccine, one case 1 week after vaccine). The patient with coughing and subfebrile body temperature two weeks after the first vaccine was seen at our HIV outpatient clinic. His nasopharyngeal swab tested negative in the diagnostic H1N1-specific and other influenza A PCR. He improved after a few days and tolerated the second vaccine well. The other three patients did not report their symptoms until the next routine contact (second vaccination or on the phone one month after the second vaccination). All blood samples collected from these four patients before and after their upper respiratory illness were retested using an influenza A and B-specific complement-fixation method and none showed a significant titer increase. These illnesses were not influenza A and therefore were not documented as vaccination failures.

At our HIV outpatient department, which regularly monitors approximately 1,000 HIV-positive individuals in a period of 6 months, only four pandemic influenza A (H1N1) infections were noticed during the study period. None of these patients had been vaccinated and all had typical symptoms such as fever, coughing, and joint pain without complications.

## Discussion

The main finding of our study is that during the pandemic HIV-positive individuals showed a limited but clear response to the unique, inactivated whole-virion, Vero-cell-derived and non-adjuvanted pandemic H1N1 vaccine.

For a vaccination to be useful, the seroprotection rate should exceed 70% in adults ≤60 years of age, according to the CHMP at the EMA [9]. Surprisingly, 70% of our HIV-positive study participants (n = 55 of 79) already had HAI antibody titers ≥1∶40 before vaccination. Unlike other influenza pandemics, during the 2009/2010 H1N1 pandemic a high percentage of the population, especially in the older age group, already showed antibody titers against this virus [20]–[22]. Similar findings can also be seen in our study, where all vaccinated HIV-positive individuals older than 60 years (n = 7) had protective HAI antibody titers of ≥1∶40, and even in younger HIV-positive individuals (≤60 years), 67% (n = 48 of 72) had protective HAI antibody titers of ≥1∶40, among whom 40% (n = 19 of 48) had HAI antibody titers >1∶40.

However our findings of a high percentage of HAI titers ≥1∶40 before vaccination are in accordance with recent HIV studies in children (46% [23]) and adults (79.6% [24]). The authors of the last mentioned study [24], in Italy, discussed several factors for these high HAI titers. Their patients were vaccinated after the beginning of the pandemic, thus exposure to or infection with influenza A (H1N1) could not be excluded. This may be relevant for our study population in Austria, a country bordering Italy. Limitations of the HAI method were also considered. Furthermore, the detection of some antibodies cross-reacting with H1N1 in HAI assay may be an additional explanation [25], [26]. Although we were not able to demonstrate an association between HAI titers before H1N1 vaccination and prior seasonal influenza vaccinations as demonstrated recently [26], ultimately the number of previously vaccinated patients was much higher (>80%), in contrast with our study (<20%). However, the majority of recent HIV studies described mainly lower HAI baseline titers [26]–[28]. Our recently published data on solid organ transplant (SOT) patients (n = 25) also showed high HAI titers: 59% had titers ≥1∶40 before vaccination (54% of 25 were patients ≤60 years) [18].

As a result of the high pre-vaccination HAI titers, we observed a seroprotection rate of 91% after the first vaccination in the age group ≤60 years, which is comparable to or better than results with adjuvanted and adjuvant-free vaccines in HIV-positive study populations [24], [27]–[29].

We found overall seroconversion rates of only 31% after the first vaccination and 41% after the second, barely meeting the licensing criteria of CHMP: >40% of subjects ≤60 years of age and >30% of subjects >60 years [9]. In the age group ≤60 years the seroconversion rate improved slightly up to 45% after the second vaccination, whereas none of the seven patients >60 years seroconverted. The seroconversion rates in our studied vaccinated SOT patients were slightly lower: 23% after the first vaccination and 37% after the second [18]. Although the majority of recent immunogenicity studies with adjuvanted pandemic H1N1 influenza vaccines in HIV-positive patients show higher seroconversion rates [30], [31], [26], some investigations have reported seroconversion rates similar to those in our study [24]. The elevated HAI titers at baseline (Table 2) may explain the notably reduced seroconversion rate; however, the rate was up to 86% in patients with lower HAI baseline titers. Whole-virion pandemic influenza vaccine is designed to provide protection to a population naïve for a novel influenza virus. This effect is well documented in our study, where a more robust immune response was observed in persons with low HAI antibody titers at baseline (Figure 3).

The third licensing criterion, the mean 2.5-fold increase GM of HAI antibody titers, was not fulfilled; nevertheless, similarly to the seroconversion rate, there was a negative correlation with baseline HAI antibody titers and the GMT ratio (Figure 3). Thus, two of the three criteria were met in our HIV-positive patients ≤60 years of age (Table 2). According to CHMP, only one of the three criteria should be met to satisfy the immunogenicity requirement, even in immunocompetent persons [9].

Local adverse events are frequent with adjuvanted vaccines, whereas the non-adjuvanted, Vero-cell-derived H1N1 influenza vaccine was well tolerated in the HIV-positive adults in our study, as also shown in healthy adults [32] and children [33]. The whole-virion adjuvant-free vaccine was designed to provide the optimal balance between immunogenicity, effectiveness, and tolerability, as it was considered that reduced tolerability might have a negative impact on vaccine uptake.

In the present study immunogenicity was measured by HAI assay and ELISA and the dissimilarity of test results was remarkable. The high number of individuals with HAI antibody titers ≥1∶40 (70%) together with the low seropositivity rate (3%) measured by virus-specific H1N1 IgG ELISA before vaccination is unusual and argues that the ELISA is poorly sensitive. Both tests demonstrated immunogenicity of the H1N1 vaccine after two doses, but it is difficult to compare the two methods. The trend of dissimilar results in the two tests was also found in our SOT patients; for example, before vaccination 59% had HAI antibody titers ≥1∶40 whereas only 18% were seropositive by ELISA [18]. A recent study by Meyer *et al.* [34] reported that the ELISA had a predictive value of only 54% for correctly identifying a vaccine responder. Dikow *et al.* [35], however, studied the immunogenicity of an adjuvanted H1N1 vaccine measured by IgG ELISA in hemodialysis patients and discussed the problem of calibrating quantitative results in the absence of an international standard. Nevertheless, because high values for arbitrary ELISA units correlate with higher quantitative amounts of antibodies, this may serve as an acceptable surrogate marker for seroconversion and seroprotection.

Only a few immunogenicity data on non-adjuvanted pandemic H1N1 vaccines in HIV-positive individuals have been published recently [28], [36], [37]. Apart from the lower protective HAI antibody baseline titers (8.6–25%) before vaccination, however, these three studies cannot be simply compared with our study. They all used a single-dose strategy with an egg-based, split-virion pandemic H1N1 vaccine containing 15 µg of hemagglutinin per vaccine dose. In our study we used a two-dose strategy with a Vero-cell-derived, whole-virion vaccine containing only 7.5 µg hemagglutinin per vaccine dose. The serological responses in patients without pandemic H1N1 protective HAI antibodies appear comparable to our study.

Our study has several limitations. The number of patients studied was small, in particular the number of pandemic H1N1-naïve individuals. Furthermore, the study was observational and non-interventional, and we did not recruit a healthy control group for direct comparison of immune responses in immunocompromised and immunocompetent persons. However, immunogenicity in healthy immunocompetent persons has been described recently: after two courses of vaccination the seroconversion rate was 49% and the cohort fulfilled all three EMA immunogenicity criteria [32]. The seroconversion rate in healthy individuals would be expected to be above the overall seroconversion rate of 41% reached by our HIV-positive patients; however, those individuals who were HIV-positive H1N1-naïve before vaccination achieved a seroconversion rate of 63% after the second dose of vaccine.

A recent study with Vero-cell-derived seasonal influenza vaccine also demonstrates that higher HAI antibody titers do not correlate with increased vaccine effectiveness: titers >1∶30 provided no additional benefit of protection [38], as also reported by other authors [10], [39]. For these reasons, differences in titers between the adjuvanted split vaccine and the whole-virion vaccine should be interpreted with caution.

In summary, our results have demonstrated that during the H1N1 pandemic of 2009/2010 use of the well-tolerated pandemic adjuvant-free influenza A (H1N1) vaccine resulted in a limited immune response in HIV-positive patients; however, the response was more powerful in the population naïve for 2009 H1N1 influenza virus before vaccination. There is a need for additional efficacy and safety studies using different dosages and interval strategies and for head-to-head comparison with adjuvanted vaccines in this risk population.

## References

[1] Dawood FS, Jain S, Finelli L, Shaw MW, Lindstrom S (2009). Emergence of a novel swine-origin influenza A (H1N1) virus in humans.. N Engl J Med.

[2] Perez-Padilla R, de la Rosa-Zamboni D, Ponce de Leon S, Hernandez M, Quinones-Falconi F (2009). Pneumonia and respiratory failure from swine-origin influenza A (H1N1) in Mexico.. N Engl J Med.

[3] Girard MP, Katz J, Pervikov Y, Palkonyay L, Kieny MP (2010). Report of the 6th meeting on the evaluation of pandemic influenza vaccines in clinical trials World Health Organization, Geneva, Switzerland, 17–18 February 2010.. Vaccine.

[4] World Health Organization (2009). Statement to the press by Director-General Dr Margaret Chan “World now at the start of 2009 influenza pandemic”. Media centre.. http://www.who.int/mediacentre/news/statements/2009/h1n1_pandemic_phase6_20090611/en/.

[5] Kaplan JE, Benson C, Holmes KH, Brooks JT, Pau A (2009). Guidelines for prevention and treatment of opportunistic infections in HIV-infected adults and adolescents: recommendations from CDC, the National Institutes of Health, and the HIV Medicine Association of the Infectious Diseases Society of America.. MMWR Recomm Rep.

[6] Kunisaki KM, Janoff EN (2009). Influenza in immunosuppressed populations: a review of infection frequency, morbidity, mortality, and vaccine responses.. Lancet Infect Dis.

[7] Evison J, Farese S, Seitz M, Uehlinger DE, Furrer H (2009). Randomized, double-blind comparative trial of subunit and virosomal influenza vaccines for immunocompromised patients.. Clin Infect Dis.

[8] Ferguson NM, Cummings DA, Fraser C, Cajka JC, Cooley PC (2006). Strategies for mitigating an influenza pandemic.. Nature.

[9] European Committee for Proprietary Medicinal Products (1997). Note for guidance on harmonisation of requirements for Influenza vaccines (CPMP/BWP/214/96). London: European Agency for the Evaluation of Medicinal Products.. http://www.ema.europa.eu/docs/en_GB/document_library/Scientific_guideline/2009/09/WC500003945.pdf.

[10] Coudeville L, Bailleux F, Riche B, Megas F, Andre P (2010). Relationship between haemagglutination-inhibiting antibody titres and clinical protection against influenza: development and application of a bayesian random-effects model.. BMC Med Res Methodol.

[11] Hobson D, Curry RL, Beare AS, Ward-Gardner A (1972). The role of serum haemagglutination-inhibiting antibody in protection against challenge infection with influenza A2 and B viruses.. J Hyg (Lond).

[12] Stephenson I, Nicholson KG, Gluck R, Mischler R, Newman RW (2003). Safety and antigenicity of whole virus and subunit influenza A/Hong Kong/1073/99 (H9N2) vaccine in healthy adults: phase I randomised trial.. Lancet.

[13] Parkman PD, Hopps HE, Rastogi SC, Meyer HMJ (1977). Summary of clinical trials of influenza virus vaccines in adults.. J Infect Dis.

[14] Lin J, Zhang J, Dong X, Fang H, Chen J (2006). Safety and immunogenicity of an inactivated adjuvanted whole-virion influenza A (H5N1) vaccine: a phase I randomised controlled trial.. Lancet.

[15] Kistner O, Crowe BA, Wodal W, Kerschbaum A, Savidis-Dacho H (2010). A whole virus pandemic influenza H1N1 vaccine is highly immunogenic and protective in active immunization and passive protection mouse models.. PLoS One.

[16] European Medicines Agency (2009). Assessment Report for Celvapan. International Nonproprietary Name: Pandemic influenza vaccine (H1N1) (whole virion, Vero cell derived, inactivated) A/California/07/2009 (H1N1)v (EMA/843807/2009).. http://www.ema.europa.eu/docs/en_GB/document_library/EPAR_-_Assessment_Report_-_Variation/human/000982/WC500059446.pdf.

[17] Johansen K, Nicoll A, Ciancio BC, Kramarz P (2009). Pandemic influenza A(H1N1) 2009 vaccines in the European Union.. Euro Surveill.

[18] Lagler H, Wenisch JM, Tobudic S, Gualdoni GA, Rodler S (2011). Pandemic influenza A H1N1 vaccine in recipients of solid organ transplants: immunogenicity and tolerability outcomes after vero cell derived, non-adjuvanted, whole-virion vaccination.. Vaccine.

[19] Mayr A, Bachmann BA, Bibrack B, Wittmann G, Mayr A, Bachmann BA, Bibrack B, Wittmann G (1977). Serologie [serology] 1^st^ ed. vol. 2.. Virologische Arbeitsmethoden [Virological methods].

[20] Miller E, Hoschler K, Hardelid P, Stanford E, Andrews N (2010). Incidence of 2009 pandemic influenza A H1N1 infection in England: a cross-sectional serological study.. Lancet.

[21] Rizzo C, Rota MC, Bella A, Alfonsi V, Declich S (2010). Cross-reactive antibody responses to the 2009 A/H1N1v influenza virus in the Italian population in the pre-pandemic period.. Vaccine.

[22] Arguedas A, Soley C, Lindert K (2010). Responses to 2009 H1N1 vaccine in children 3 to 17 years of age.. N Engl J Med.

[23] Esposito S, Tagliaferri L, Daleno C, Valzano A, Picciolli I (2011). Pandemic influenza A/H1N1 vaccine administered sequentially or simultaneously with seasonal influenza vaccine to HIV-infected children and adolescents.. Vaccine.

[24] Kajaste-Rudnitski A, Galli L, Nozza S, Tambussi G, Di Pietro A (2011). Induction of protective antibody response by MF59-adjuvanted 2009 pandemic A/H1N1v influenza vaccine in HIV-1-infected individuals.. AIDS.

[25] Hancock K, Veguilla V, Lu X, Zhong W, Butler EN (2009). Cross-reactive antibody responses to the 2009 pandemic H1N1 influenza virus.. N Engl J Med.

[26] Soonawala D, Rimmelzwaan GF, Gelinck LB, Visser LG, Kroon FP (2011). Response to 2009 Pandemic Influenza A (H1N1) Vaccine in HIV-Infected Patients and the Influence of Prior Seasonal Influenza Vaccination.. PLoS One.

[27] Bickel M, Wieters I, Khaykin P, Nisius G, Haberl A (2010). Low rate of seroconversion after vaccination with a split virion, adjuvanted pandemic H1N1 influenza vaccine in HIV-1-infected patients.. AIDS.

[28] Tebas P, Frank I, Lewis M, Quinn J, Zifchak L (2010). Poor immunogenicity of the H1N1 2009 vaccine in well controlled HIV-infected individuals.. AIDS.

[29] Orlando G, Pariani E, Mazza F, Tanzi E, Meraviglia P (2010). Pandemic influenza vaccine in adult HIV-1-infected patients.. AIDS.

[30] Manuel O, Pascual M, Hoschler K, Giulieri S, Alves D (2011). Humoral response to the influenza A H1N1/09 monovalent AS03-adjuvanted vaccine in immunocompromised patients.. Clin Infect Dis.

[31] Crum-Cianflone NF, Eberly LE, Duplessis C, Maguire J, Ganesan A (2011). Immunogenicity of a monovalent 2009 influenza A (H1N1) vaccine in an immunocompromised population: a prospective study comparing HIV-infected adults with HIV-uninfected adults.. Clin Infect Dis.

[32] Nicholson KG, Abrams KR, Batham S, Clark TW, Hoschler K (2011). Immunogenicity and safety of a two-dose schedule of whole-virion and AS03A-adjuvanted 2009 influenza A (H1N1) vaccines: a randomised, multicentre, age-stratified, head-to-head trial.. Lancet Infect Dis.

[33] Waddington CS, Walker WT, Oeser C, Reiner A, John T (2010). Safety and immunogenicity of AS03B adjuvanted split virion versus non-adjuvanted whole virion H1N1 influenza vaccine in UK children aged 6 months-12 years: open label, randomised, parallel group, multicentre study.. BMJ.

[34] Meyer S, Adam M, Schweiger B, Ilchmann C, Eulenburg C (2011). Antibody Response After a Single Dose of an AS03-Adjuvanted Split-Virion Influenza A (H1N1) Vaccine in Heart Transplant Recipients.. Transplantation.

[35] Dikow R, Eckerle I, Ksoll-Rudek D, Hampel H, Schwenger V (2011). Immunogenicity and Efficacy in Hemodialysis Patients of an AS03(A)-Adjuvanted Vaccine for 2009 Pandemic Influenza A(H1N1): A Nonrandomized Trial.. Am J Kidney Dis.

[36] Ho J, Moir S, Wang W, Posada JG, Gu W (2011). Enhancing effects of adjuvanted 2009 pandemic H1N1 influenza A vaccine on memory B-cell responses in HIV-infected individuals.. AIDS.

[37] Miraglia JL, Abdala E, Hoff PM, Luiz AM, Oliveira DS (2011). Immunogenicity and reactogenicity of 2009 influenza A (H1N1) inactivated monovalent non-adjuvanted vaccine in elderly and immunocompromised patients.. PLoS One.

[38] Barrett PN, Berezuk G, Fritsch S, Aichinger G, Hart MK (2011). Efficacy, safety, and immunogenicity of a Vero-cell-culture-derived trivalent influenza vaccine: a multicentre, double-blind, randomised, placebo-controlled trial.. Lancet.

[39] de Jong JC, Palache AM, Beyer WE, Rimmelzwaan GF, Boon AC (2003). Haemagglutination-inhibiting antibody to influenza virus.. Dev Biol (Basel).

